# Free Innervated Gracilis Muscular Flap for Functional Abdominal Wall Reconstruction Following Desmoid Tumor Resection: A Case Report and Literature Review

**DOI:** 10.1002/micr.70306

**Published:** 2026-10-15

**Authors:** Efstathios G. Lykoudis, Marios Papadakis, Ilias Stylianos K. Iliadis, George E. Lykoudis, Dimitra V. Peristeri, Ioannis Dalianoudis

**Affiliations:** ^1^ Department of Plastic and Burn Surgery Ioannina University School of Medicine Ioannina Greece; ^2^ Department of Radiology Ioannina University School of Medicine Ioannina Greece

**Keywords:** abdominal wall, defect, desmoid tumor, gracilis, reconstruction

## Abstract

Desmoid tumors (DTs) are rare, benign, locally aggressive myofibroblastic neoplasms. Complete resection with negative margins remains the standard treatment despite their lack of metastatic potential. As DT commonly involve the anterior abdominal wall, their excision may result in complex defects requiring advanced reconstruction. We herein present the first functional single‐stage reconstruction of an abdominal wall defect with the use of a free innervated gracilis muscle flap transfer. A 29‐year‐old woman, presented with a palpable tumor on the left‐side of her abdominal wall. She underwent surgery and on biopsy, the tumor was diagnosed as a DT. The patient underwent wide resection through an abdominoplasty approach, which resulted in a rectus abdominis defect involving the anterior rectus abdominis sheath. The 8 × 7cm‐sized defect was reconstructed with free transfer of a neurotized gracilis muscle flap, anastomosed to the deep inferior epigastric vessels, whereas, the anterior branch of the obturator nerve was coapted to the ninth intercostal nerve. Finally, a monofilament polypropylene mesh was used for reconstruction of the missing anterior rectus sheath. Ever since and during a follow‐up of 5 years, electromyographic examinations have demonstrated excellent re‐innervation of the gracilis muscle flap. The patient underwent regular follow‐up examinations, including CT imaging at 1, 2, and 5 years postoperatively, with no evidence of abdominal wall protrusion or tumor recurrence. This case demonstrates that the free innervated gracilis flap can provide a safe and reliable reconstructive option for complex abdominal wall defects following oncologic resection.

## 1 Introduction

Desmoid tumors (DTs) are a rare entity of benign, locally aggressive myofibroblastic neoplasms, classified as deep fibromatoses. Due to their extensive local infiltration, they frequently recur. Although DT lack a metastatic potential, complete resection with free margins is considered as the standard treatment (Harati et al. 2017). Given that, DTs most often occur in the anterior abdomen, particularly at any musculo‐aponeurotic junction, their resection may result in composite defects, affecting the structural integrity, strength and contour of the anterior abdominal wall, often leading to loss of function, abdominal laxity and visceral herniation (Ver Halen et al. 2015; Fiore et al. 2016).

Over the years, various methods have been suggested for reconstruction of those defects (Ver Halen et al. 2015). While local and regional flaps are effective for soft tissue coverage, they do not re‐establish the dynamic support required for optimal abdominal wall function. Restoration of functional integrity requires innervated muscle transfer, such as pedicled tensor fascia lata or rectus femoris flaps for lower abdominal wall defects. Dynamic abdominal wall reconstruction is commonly achieved using pedicled innervated tensor fascia lata or rectus femoris flaps, which provide vascularized tissue and functional muscle transfer. However, their use is limited by arc of rotation, donor‐site morbidity, and sacrifice of major lower extremity muscles. Free functional muscle transfer overcomes these limitations and enables reconstruction of extensive or anatomically challenging defects (Lykoudis et al. 2005). The free latissimus dorsi flap has been described for this purpose because of its large surface area and reliable neurovascular pedicle but requires intraoperative repositioning, is relatively bulky, and is associated with donor‐site morbidity and potential shoulder dysfunction (Ninković et al. 1998; Malheiro et al. 2007). In contrast, the gracilis muscle offers a versatile alternative, with consistent vascular and neural anatomy, ease of harvest, minimal donor‐site functional deficit, and suitable muscle excursion, making it an attractive option for dynamic abdominal wall reconstruction.

Herein, we present a case of near‐total loss of the left rectus abdominis muscle and a part of the overlying anterior fascia, due to resection of a DT located in the anterior abdominal wall. Functional single‐stage reconstruction of the resulted defect was provided with the use of a neurotized gracilis muscle free flap transfer. To the best of our knowledge, this is the first report of functional abdominal wall reconstruction by means of a free innervated muscular gracilis flap following DT resection.

## 2 Case Report

A 29‐year‐old woman was referred to our hospital with a palpable tumor on the left‐side of her abdominal wall. The mass was infiltrating the ipsilateral rectus abdominis muscle and was also in close contact with the overlying anterior rectus sheath. On biopsy, the tumor was diagnosed as a DT. The case was presented in the hospital's tumor board and a wide surgical excision has been recommended. Wide resection through an abdominoplasty approach, resulted in a rectus abdominis defect measuring 15 cm in length, as well as, a defect of the anterior rectus abdominis sheath approximately 8 × 7 cm (Figure 1). Tumor excision was followed by immediate reconstruction with free transfer of a neurotized gracilis muscle flap. A segment of the gracilis, equal in length to the missing rectus abdominis, was harvested from the right thigh and carefully sutured to the proximal and distal stumps of rectus abdominis muscle. The deep inferior epigastric vessels were dissected and anastomosed to the vascular pedicle of the gracilis, whereas, the anterior branch of the obturator nerve innervating the gracilis was carefully coapted to the already dissected, during tumor resection, 9th intercostal nerve (Figure 2).

**FIGURE 1:**
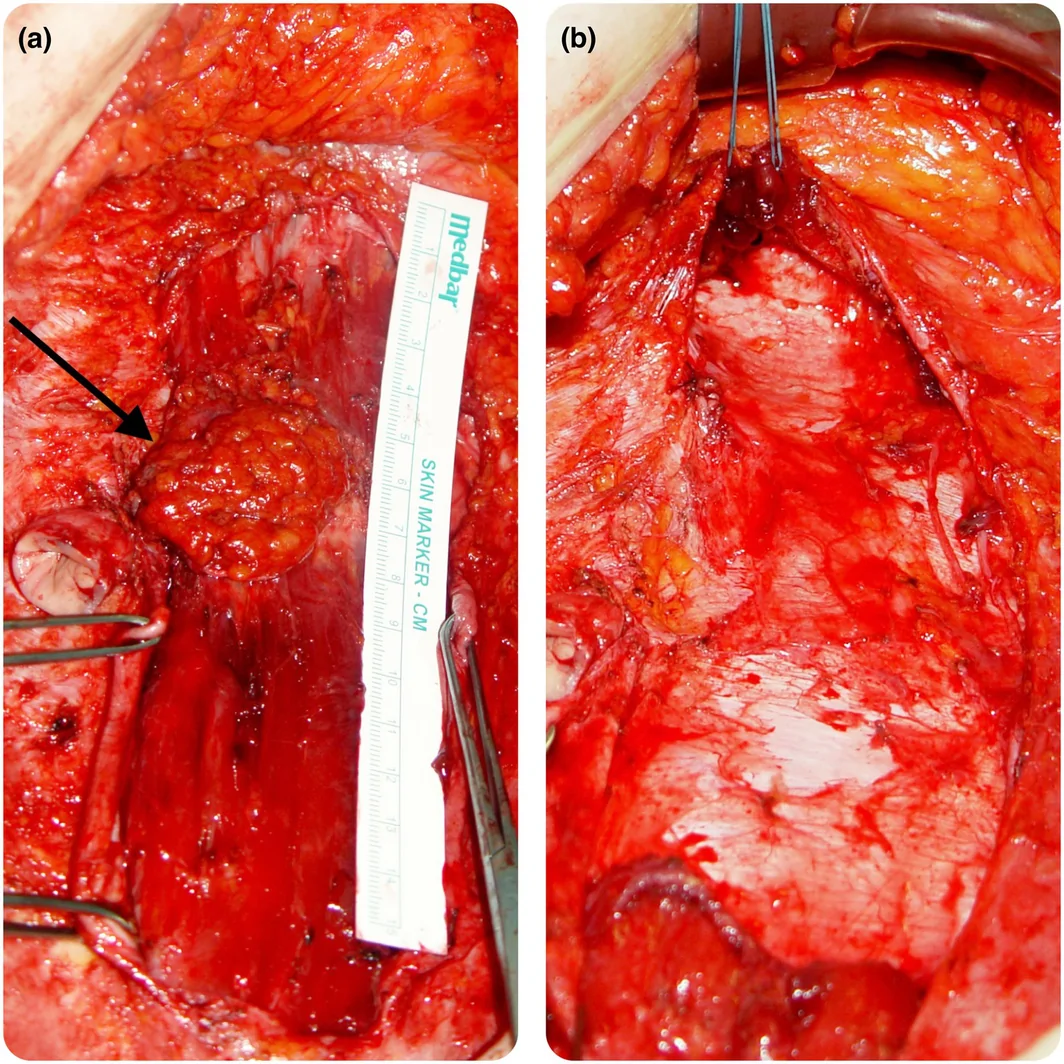
Desmoid tumor (arrow) along with the segment of the rectus abdominis to be resected (a). The defect inflicted by the subtotal rectus abdominis excision and tumor resection (b).

**FIGURE 2:**
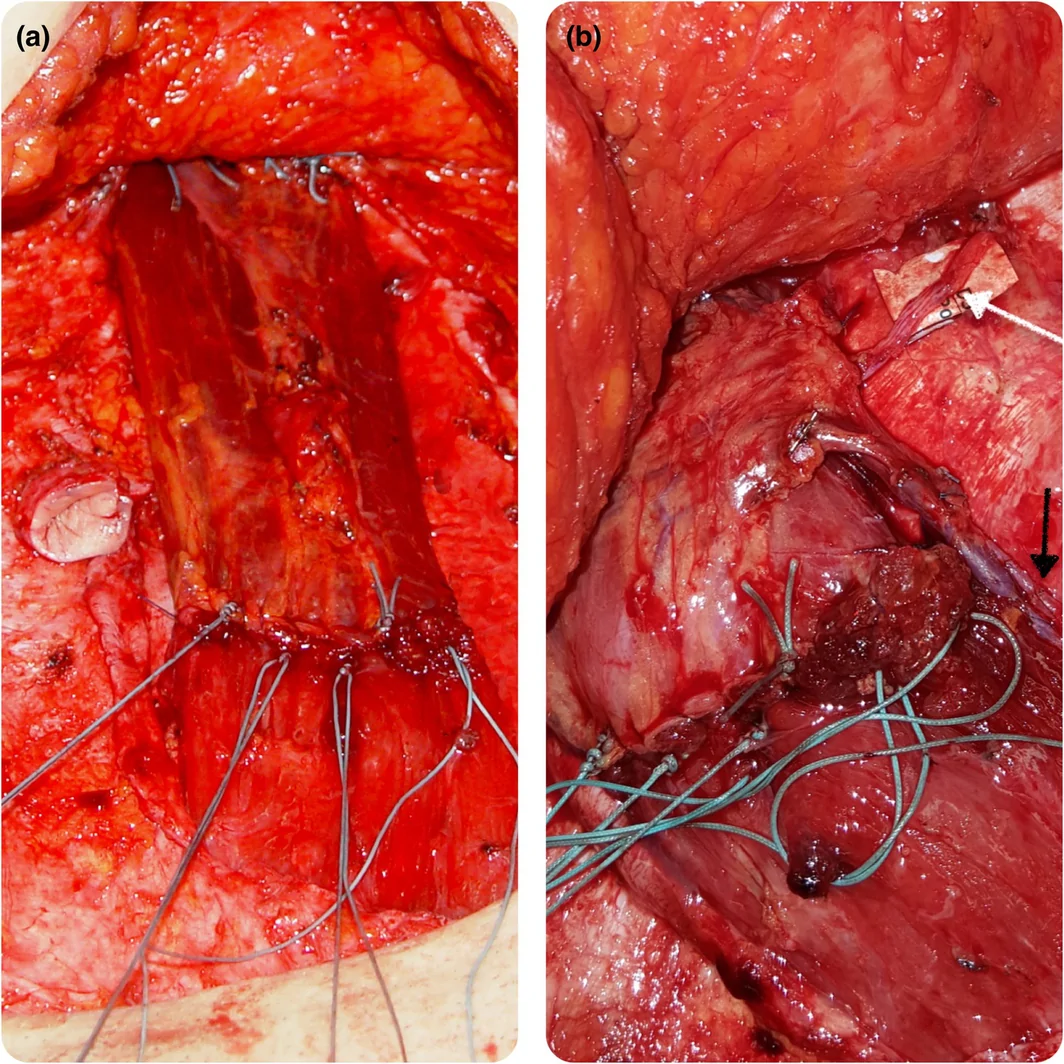
Free gracilis flap inset to the recipient site (a). Coaptation of the anterior obturator nerve to the 9th intercostal nerve (white arrow), following completion of the arterial and venous anastomoses (black arrow) (b).

The ninth intercostal nerve was selected as the recipient nerve in order to achieve a more physiologically aligned reinnervation of the transferred gracilis muscle. Intraoperatively, it was identified at the lateral border of the rectus abdominis muscle. No intraoperative nerve stimulation or mapping was required. The nerve was transected laterally to allow tension‐free coaptation to the anterior branch of the obturator nerve of the gracilis flap. Care was taken to preserve surrounding structures. Finally, a piece of knitted monofilament polypropylene mesh was used for reconstruction of the missing anterior rectus sheath (Figure 3). The mesh was positioned in a way that did not restrict the mobility of the muscle, allowing unimpeded contraction of the neurotized flap, and was secured with interrupted non‐absorbable sutures to the surrounding fascial edges, ensuring stable fixation and adequate tension distribution across the defect.

**FIGURE 3:**
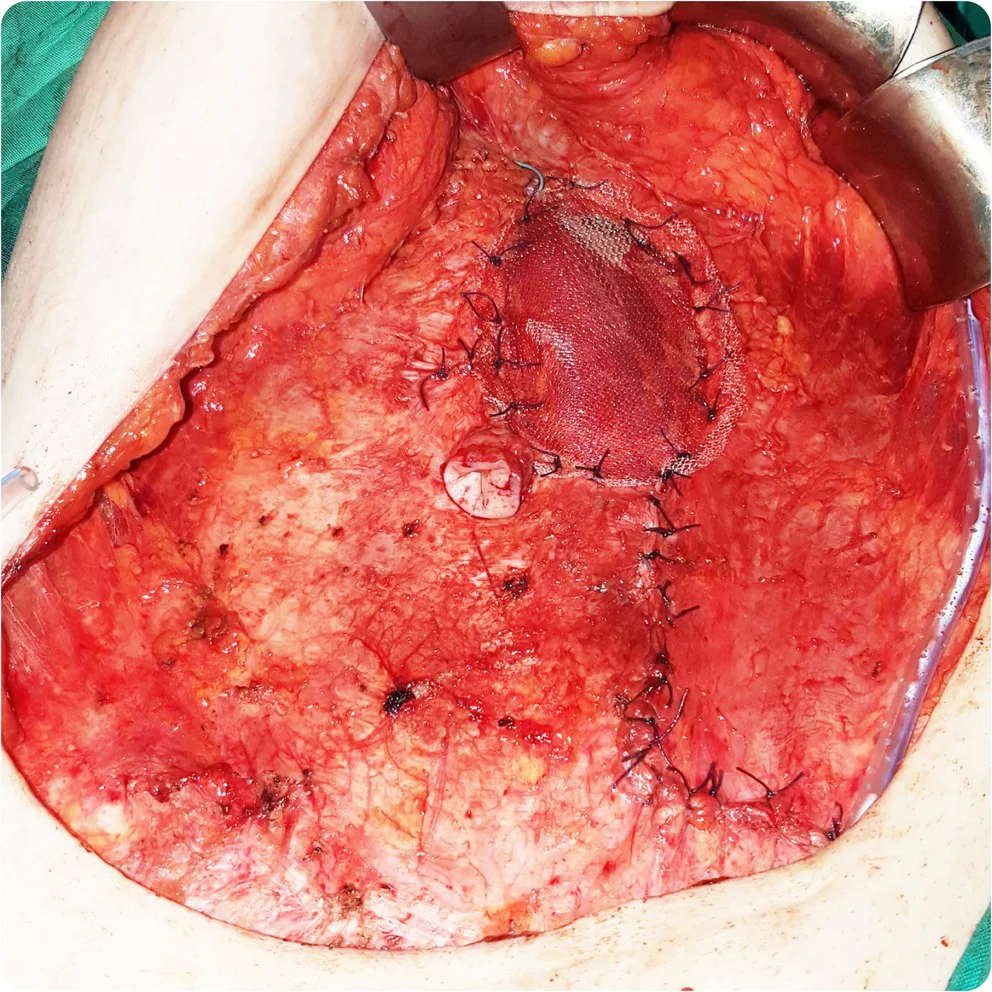
The synthetic mesh sutured over the free gracilis flap to reconstruct the missing anterior rectus abdominis sheath.

The postoperative course was uneventful. The patient was allowed to walk with an elastic abdominal binder 1 week after surgery, which the patient continued to wear for 8 months postoperatively. The patient was discharged on the 12th postoperative day. The patient was also provided a slow‐pulse stimulator (“The Liberson,” J.D. Medical, California, USA) following thorough education on its proper utilization, to prevent atrophy of the transferred gracilis muscle. Stimulation was delivered at low frequency (20 Hz), with pulse durations in the range of 200–300 μs. Sessions were conducted for approximately 30–60 min, adjusted according to patient tolerance and muscle response. The stimulator was used on a daily base for 1 year.

Electromyographic findings proved voluntary contraction of the transferred muscle 7 months after surgery. Ever since and during a follow‐up of 5 years, electromyographic examinations have demonstrated excellent reinnervation of the gracilis muscle flap. The patient underwent regular follow‐up examinations including CT imaging conducted at intervals of 1 year, 2 years, and 5 years postoperatively, where neither abdominal protrusion nor tumor recurrence were observed (Figure 4). No clinically evident donor‐site morbidity related to intercostal nerve harvest (such as abdominal wall weakness or sensory deficit) was observed during follow up. Currently, the patient is able to do sit‐ups easily without fatigue, and has returned to her normal occupational and daily activities without restriction.

**FIGURE 4:**
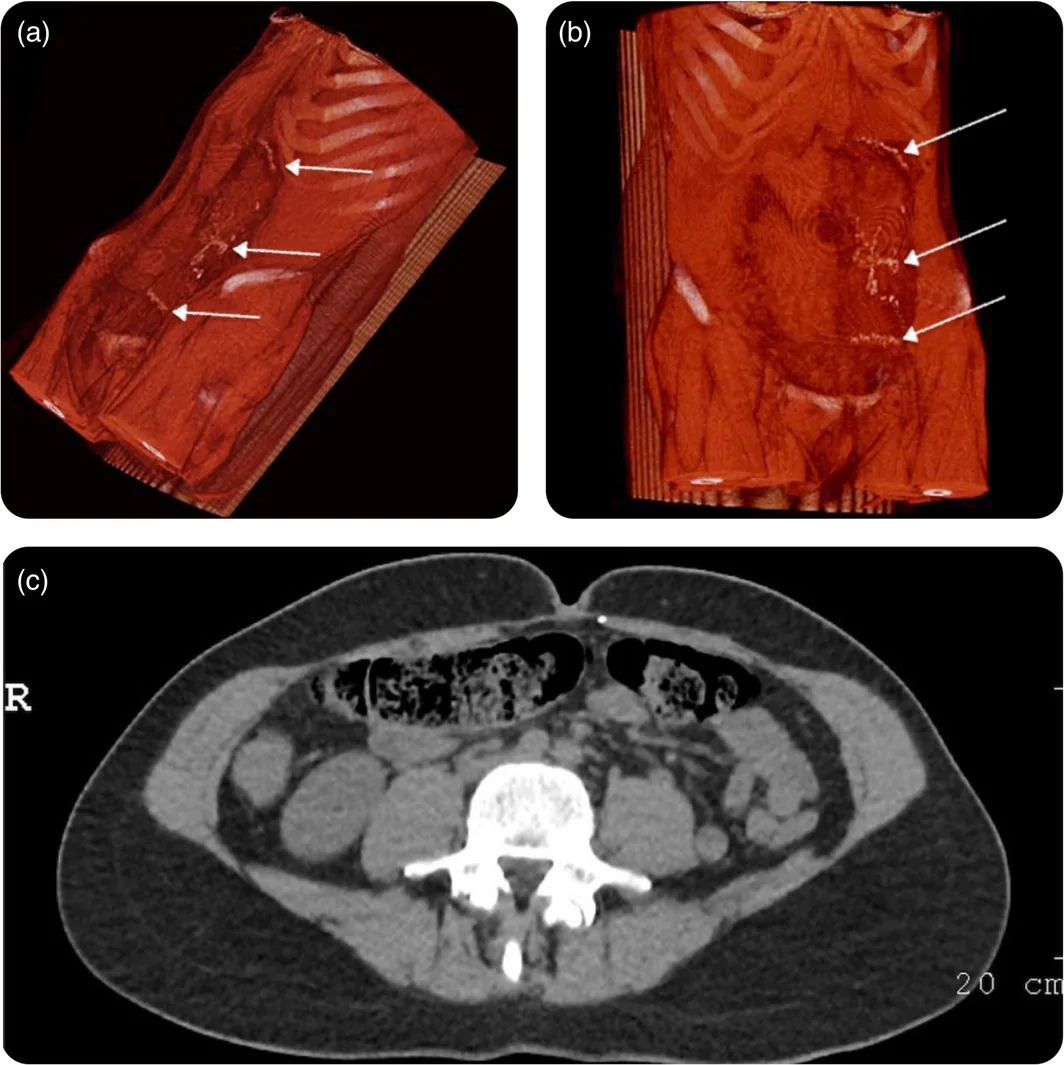
Five year follow up CT imaging and CT scan 3D reconstruction of the abdominal wall. The upper and lower arrows represent the proximal and distal suture lines of the gracilis to the corresponding remnants of the rectus abdominis. The middle arrow corresponds to the synthetic mesh over the gracilis muscle (a, b). Axial CT plane of the healthy rectus abdominis to the right (R) and the transferred gracilis muscle to the left at the level of the umbilicus (c).

## 3 Discussion

DT belongs to a group of rare soft tissue neoplasms, characterized by infiltrative growth and a tendency toward local recurrence, though unable to metastasize. The histopathologic confirmation of DT is mandatory before initiating treatment and can be readily established with needle core biopsies. Immediate surgery is no more the standard treatment of DT, since regressions of up to 28% have been recorded (Fiore et al. 2016). Therefore, initial observation under active surveillance, up to 2 years, can be proposed, provided that the patient is asymptomatic and tumor size remains stable. In case of progressing DT located in the abdominal wall, radical surgical resection to clear margins is the treatment of choice, provided that the expected surgical morbidity is limited. Conventional reconstruction of the resected abdominal muscles (rectus or/and obliques) is usually performed using a synthetic mesh, whereas, the use of a flap is added for the missing abdominal skin. However, the abdominal wall function is significantly impaired. Advances in microsurgical techniques coupled with the increased knowledge of vascular anatomy, can nowadays provide, in a single stage, high‐level functional reconstructions with the use of flaps tailored to individual defects (Lykoudis et al. 2005).

A thorough literature review for functional abdominal wall reconstruction (Table 1), revealed six separate types of functional muscle or musculocutaneous flap transfers, that is, the free latissimus dorsi flap (Ninković et al. 1998; Malheiro et al. 2007), the tensor fascia lata musculocutaneous flap (Sasaki et al. 1998; Wong et al. 2009; Chalfoun et al. 2012; Grinsell et al. 2012), the rectus femoris (Koshima et al. 1999, 2003), and the free (and pedicled) innervated vastus lateralis along with the anterolateral thigh flap (Iida et al. 2013; Fukui et al. 2016; Vranckx et al. 2015; DI Summa et al. 2019). All these flaps are associated with severe donor‐site morbidity, the latter necessitating sacrificing of a vital thigh muscle, leading to a reduction in lower limb muscular power.

**TABLE 1:** Functional abdominal wall reconstruction in the literature.

Flaps									
References	Number	Etiology	Reconstruction type	Defect location	Defect composition	Flap type	Flap composition	Innervation	Vascular supply
Venugopalan (1980)	20	Postoperative defect Incisional hernia	Delayed	Middline lower abdomen	Hernia—wall reinforcement	Pedicled G (20)	M	Anterior branch of obturator nerve	Medial circumflex femoral artery
Ninković et al. (1998)	2	Accidents	Delayed	Hemia abdominal defect Lower lateral and central abdomen	Full thickness	Free LD (2)	M	Intercostal	Superior epigastric (1) Deep inferior epigastric (1)
Sasaki et al. (1998)	4	Malignancy	Immediate	Middle lateral abdomen Middle central abdomen Middle upper abdomen Lower central abdomen	Full thickness	Free combined TFL and ALT (4)	MC	Intercostal	Inferior epigastric and deep iliac circumflex
Koshima et al. (1999)	1	Postoperative defect	Delayed	Middle lower abdomen	Hernia—wall reinforcement	Free RF (1)	M	Intercostal	Lateral circumflex femoral
Koshima et al. (2003)	1	Postoperative defect	Delayed	Middle lower abdomen	Hernia—wall reinforcement	Free RF (1)	MC	Intact femoral nerve branch (pedicled)	Omental vessels
Malheiro et al. (2007)	1	Acute abdomen	Delayed	Upper central abdomen	Abdominal bulging—full thickness wall reinforcement	Free LD (1)	MC	Intercostal	Deep inferior epigastric
Wong et al. (2009)	2	Postoperative Trauma	Delayed	Lateral abdomen Middline abdomen	Full thickness	Free TFL (1) ALT (1)	MC	Intercostal	Femoral artery using great saphenous vein graft
Chalfoun et al. (2012)	2	Trauma Unknown	Delayed	Lower central abdomen Not reported	Full thickness	Free TFL (2)	OMF	Intercostal	Deep inferior epigastric
Grinsell et al. (2012)	2	Malignancy	unknown	Lateral abdomen	Full thickness	Free TFL (2)	Not reported	Not reported	Not reported
Iida et al. (2013)	2	Malignancy	Immediate	Right hypochondriac region (2)	Full thickness	Free combined VL and ALT (2)	MC	Intercostal	Deep inferior epigastric
Vranckx et al. (2015)	15	Chronically infected abdominal defects	Delayed	Middline	Hernia—wall reinforcement	Pedicled VL and ALT (15)	MC	Femoral nerve branch	Descending branch of deep femoral artery
Fukui et al. (2016)	2	Malignancy	Immediate	lower central abdomen	Full thickness	Pedicled VL and ALT (2)	MC	Femoral nerve branch	Descending branch of deep femoral artery
Mujadzic et al. (2018)	1	Traumatic	Immediate	Lateral abdomen	Full thickness	Pedicled G (1)	M	Anterior branch of obturator nerve	Medial circumflex artery
DI Summa et al. (2019)	4	Traumatic (1) DT (2) Eventration (1)	Immediate (1) Delayed (3)	Middline supraumbical and infraumbilical	Hernia—wall reinforcement and primary full thickness defects	Free combined VL and ALT (4)	MC	Intercostal	Deep inferior epigastric
DI Summa et al. (2019)	10	Malignancy (5) Eventration (3) Enterocutaneous fistula (2)	Immediate (7) Delayed (3)	Middline supraumbical and infraumbilical	Hernia—wall reinforcement and primary full thickness defects	Pedicled combined VL and ALT (10)	MC	Femoral nerve branch	Lateral femoral circumflex
Rossetto et al. (2010)	3	Malignancy	Immediate	Lower abdomen	Full thickness	Free Gracilis	MC	Iliohypogastric nerve	Deep inferior epigastric or gastroepiploic vessels
Carrara et al. (2024)	1	Prune‐belly syndrome	Delayed (two‐stage)	Anterior abdomen	Abdominal wall muscle agenesia	Pedicled gracilis bilaterally	M	Not applicable	Superior neurovascular pedicle

Abbreviations: ALT, anterolateral thigh; G, gracilis; LD, latissimus dorsi; M, muscular; MC, myocutaneous; OMF, osteomyofascial; RF, rectus femoris; TFL, tensor fascia lata; VL, vastus lateralis.

Mujadzic et al. have used a pedicled gracilis flap for the reconstruction of the abdominal wall after a traffic‐related injury (Mujadzic et al. 2018). Finally, just recently, Fernández‐Garrido et al. reported the use of free innervated gracilis flaps for functional abdominal wall reconstruction, with nerve coaptation performed to the iliohypogastric nerve (Fernández‐Garrido et al. 2023). On the contrary, we performed coaptation of the anterior branch of the obturator nerve to the 9th intercostal nerve. This distinction is functionally relevant. The rectus abdominis muscle is primarily innervated by the intercostal nerves (T7–T11) and the subcostal nerve (T12). Therefore, coaptation to an intercostal nerve may provide a more physiologically aligned reinnervation, potentially allowing for improved synchronization with the native abdominal wall musculature. In this context, our approach aims to achieve a closer “like‐for‐like” functional reconstruction.

In our case, the wide excision of the DT, resulted in a subtotal defect of the rectus abdominis muscle, coupled with a smaller defect of the anterior rectus sheath, sparing however, the overlying skin. Low‐frequency stimulation was chosen to promote physiologic, fatigue‐resistant muscle contractions, enhance local perfusion, and support neuromuscular junction maturation during reinnervation. The protocol is applied in a semi‐standardized manner based on established FES principles, but is always individualized empirically according to clinical progress, patient tolerance, and observed contractile response of the transferred muscle. We followed up the patient and could confirm functional recovery 5 years after surgery, however this is supported primarily by EMG findings and clinical observation rather than standardized outcome measures, which is a limitation of our study.

Given that loss of rectus abdominis is associated with weakening of abdominal wall strength, as well as up to 5.9% incidence of abdominal wall hernia and 17.3% incidence of abdominal bulging (Rossetto et al. 2010), it is our belief that, when feasible, it is worth performing functional reconstruction in young and active patients. Our preference toward a free muscular gracilis flap is based on the fact that the gracilis muscle is expendable, exhibits consistent anatomy and its harvesting is associated with minimal donor‐site morbidity. Most importantly, its shape and dimensions align closely to those of rectus abdominis and can be harvested with a skin paddle, as a musculocutaneous flap, thus enabling its application in cases of composite abdominal defects. Finally, its harvesting results in an inconspicuous donor site scar, which is easily concealed. Meticulous planning is imperative in this regard. For centrally located defects, a gracilis muscle flap with a longitudinal skin paddle is the preferred approach for harvesting and transfer. In contrast, for defects in the lower abdominal wall, a transverse cutaneous paddle emerges as a more suitable and effective option.

We conclude that the free innervated gracilis flap may be a safe and reliable method for abdominal wall reconstruction following oncological resections in active patients wishing long‐term stability and a more coordinated contraction pattern.

## Author Contributions

**Efstathios G. Lykoudis:** conceptualization, data collection, literature review, and manuscript drafting. **Marios Papadakis:** literature review and manuscript drafting. **Ilias Stylianos K. Iliadis**, **George E. Lykoudis**, and **Dimitra V. Peristeri:** data collection and literature review. **Ioannis Dalianoudis:** literature review, supervision. All authors reviewed and approved the final version.

## Funding

The authors have nothing to report.

## Ethics Statement

The authors have nothing to report.

## Consent

Informed consent was obtained from the patient.

## Conflicts of Interest

The authors declare no conflicts of interest.

## Data Availability

Data sharing not applicable to this article as no datasets were generated or analyzed during the current study.
